# Positive and Negative Affect influences Attitudes Toward Aging

**DOI:** 10.1093/geroni/igab046.2314

**Published:** 2021-12-17

**Authors:** R INTRIERI, Paige Goodwin

**Affiliations:** Western Illinois University, Macomb, Illinois, United States

## Abstract

The Aging Semantic Differential (ASD; Rosencranz & McNevin, 1969) is one of the most widely used measures in the aging literature to measure attitudes rather than knowledge or beliefs about aging. Originally 32-items the ASD has been reduced through careful factor analysis to 20-items representing 4 factors representing: Instrumentality, Autonomy, Acceptability, and Integrity. Latent summary scores were created for each factor, with lower scores representing more positive attitude toward older adults. Despite its widespread use there are no published studies that examine the relationships between the four ASD factors and Positive and Negative affect. Positive and Negative affect are related to and represent the core aspects of Extraversion and Neuroticism. The prime objective of this study was to assess the relationships between Positive and Negative affect and the four ASD factors. The sample comprises 1189 undergraduate participants with a mean age of 22.02 (SD=6.27). The sample included 611 men and 578 women. Results showed the path model fitted the data well (CFI = 953, TLI = .944, RMSEA = 0.066, SRMR = 0.035). Positive affect was significantly related to Instrumentality, Acceptability, and Integrity (β= -0.073, (SE= 0.034); p=0.034; β= --0.141 (SE= 0.033), p=0.0001; β= -0.146 (SE= 0.032), p=0.0001). These results show that higher positive affect was related to more positive beliefs about Instrumentality, Acceptability, and Integrity. Negative affect was significantly related to Integrity (β= 0.079, (SE= 0.032); p=0.012) indicating that greater negative affect was related to more negative beliefs about bodily integrity.

